# Portrayal of electronic cigarettes on YouTube

**DOI:** 10.1186/1471-2458-14-1028

**Published:** 2014-10-03

**Authors:** Chuan Luo, Xiaolong Zheng, Daniel Dajun Zeng, Scott Leischow

**Affiliations:** Institute of Automation, Chinese Academy of Sciences, The State Key Laboratory of Management and Control for Complex Systems, Beijing, China; Department of Management Information Systems, University of Arizona, Tucson, AZ 85721 USA; Mayo Clinic, Scottsdale, Arizona USA

**Keywords:** Tobacco control, Electronic cigarettes, Social media, YouTube

## Abstract

**Background:**

As the most popular video sharing website in the world, YouTube has the potential to reach and influence a huge audience. This study aims to gain a systematic understanding of what e-cigarette messages people are being exposed to on YouTube by assessing the quantity, portrayal and reach of e-cigarette videos.

**Methods:**

Researchers identified the top 20 search results on YouTube by relevance and view count for the following search terms: “electronic cigarettes”, “e-cigarettes”, “ecigarettes”, “ecigs”, “smoking electronic cigarettes”, “smoking e-cigarettes”, “smoking ecigarettes”, “smoking ecigs”. A sample of 196 unique videos was coded for overall portrayal and genre. Main topics covered in e-cigarette videos were recorded and video statistics and viewer demographic information were documented.

**Results:**

Among the 196 unique videos, 94% (n = 185) were “pro” to e-cigarettes and 4% (n = 8) were neutral, while there were only 2% (n = 3) that were “anti” to e-cigarettes. The top 3 most prevalent genres of videos were advertisement, user sharing and product review. 84.3% of “pro” videos contained Web links for e-cigarette purchase. 71.4% of “pro” videos claimed that e-cigarettes were healthier than conventional cigarettes. Audience was primarily from the United States, the United Kingdom and Canada and “pro” e-cigarette videos were watched more frequently and rated much more favorably than “anti” ones.

**Conclusions:**

The vast majority of information on YouTube about e-cigarettes promoted their use and depicted the use of e-cigarettes as socially acceptable. It is critical to develop appropriate health campaigns to inform e-cigarette consumers of potential harms associated with e-cigarette use.

## Background

An electronic cigarette, also known as an e-cigarette, is a battery-powered device that provides inhaled doses of vaporized nicotine solution, which simulates the act of tobacco smoking and looks like a real cigarette. Recent years have witnessed tremendous growth in the e-cigarette marketplace. E-cigarettes were introduced to the Chinese market in 2004 and to the US market in 2007. Since 2008, US e-cigarette sales have been doubling every year, expected to reach $1 billion in 2013 [1, 2]. Although health benefits and possible adverse effects of e-cigarettes are still unsettled by the scientific community and there have been heated discussions as to regulatory approaches to e-cigarettes, it is undeniable that e-cigarettes are here to stay and could experience sustained growth as this line of product is quickly entering the public’s consciousness. A recent U.S. survey found that in 2011, 57.9% of the general public heard about e-cigarettes, rising dramatically from 40.9% in 2010 [3]. According to Google Trends, the volume of Web searches on “electronic cigarettes” has grown 50 times in the past 4 years [4]. Mobile applications dedicated to enabling social networking among e-cigarette consumers, referred to as “social smoking,” have been introduced (e.g., Blu Cigs Smart Pack). Many mainstream tobacco companies have acquired e-cigarette vendors. E-cigarette product promotional campaigns have been conducted on multiple mainstream marketing channels. Still a niche market representing a fraction of the tobacco product sales, this disruptive technology promises to reshape the tobacco industry and perhaps even pharmaceutical companies that market nicotine replacement products, prompting significant research interests and regulatory concerns.

Although the e-cigarette research literature is growing steadily, there are significant gaps to be filled. For consumers, healthcare providers, and regulators alike, a much better understanding of e-cigarettes’ health risks, efficacy for smoking reduction and cessation, and marketing efforts and messaging practice by e-cigarette vendors, is in critical demand. Although some e-cigarette companies market e-cigarette as a smoking cessation product, clinical studies have been inconclusive [5]. Some studies indicated that e-cigarette use led to substantially decreased cigarette consumption among smokers [6]. At the same time, there are concerns that e-cigarettes could become “bridge products” for use in places prohibiting smoking or starter products, attracting young people or former smokers to smoking [7]. In the absence of regulatory control, marketing of e-cigarette products in both mass and social media, has been prevalent, leading to confusion and raising concerns about the sustained effectiveness of tobacco control.

Founded in February 2005, YouTube allows billions of people to discover, watch and share originally-created videos. As the most popular video sharing website in the world, more than 1 billion unique users visit YouTube each month [8]. There are many publicly available videos that purposely promote tobacco use on YouTube [9]. Messages embedded in YouTube have the potential to influence tobacco-related attitudes, beliefs and behaviors. Social media websites like YouTube have recently become a critical platform for health surveillance [10] and social intelligence [11]. Taking YouTube as a data source, previous researchers have studied information on ‘smoking’ [12], smoking cessation [13], smoking imagery associated with cigarettes [14], smokeless tobacco [15] and little cigars [16]. Although there is a study which mined data on usage of e-cigarettes from YouTube videos [17], they didn’t examine the portrayal of e-cigarette content.

Despite the growing literature on the portrayal of tobacco on YouTube, there are no published studies to date that have systematically assessed e-cigarette content on YouTube. Given the potential that YouTube has to promote e-cigarette use through user-generated content or covert advertising, this study aims to gain a better understanding of what e-cigarette messages people are being exposed to on YouTube.

## Methods

### Data collection

We sampled e-cigarette related videos on YouTube for study purpose in line with several previous related studies [15, 16]. Using YouTube’s search engine we conducted searches for e-cigarette videos on February 3, 2013. The sample of YouTube videos was selected from the top search results for the following search terms: “electronic cigarettes”, “e-cigarettes”, “ecigarettes”, “ecigs”, “smoking electronic cigarettes”, “smoking e-cigarettes”, “smoking ecigarettes”, “smoking ecigs”. Through a Google Trends analysis we found that there is a higher proportion of web traffic searching for these search terms than other terms. In addition, a preliminary search on YouTube also indicated that these terms would pull up more relevant and popular e-cigarette videos than other terms. As a result, these eight search terms were chosen because they are the most frequently used terms for e-cigarettes and they can cover both “pro” and “anti” e-cigarette videos.

Two searches were conducted for each term: (a) “by relevance” and (b) “by view count”. These two kinds of search strategy were chosen to mimic the typical user behavior by using the default search strategy (searching “by relevance”) as well as capture the most popular videos (searching “by view count”). Based on insight on user browsing behavior gained from previous studies [18] on a several Internet search engines indicating that the majority of people will only click on the first page of search results, we assume that few users would watch more than 20 videos since the first page of YouTube search results contains 20 videos. As a result, the sample was limited to the top 20 videos for each search. The initial sample included 320 videos in total (20 videos for each of the eight search terms and each of the two search strategies).

After obtaining the initial sample, we then eliminated videos that were not relevant to e-cigarettes and videos that were duplicates (n = 124). Videos were considered not relevant to e-cigarettes if they didn’t feature e-cigarettes or there was only a brief mention or image of e-cigarettes. Duplicate videos that appeared more than once by using different search terms and search strategies were also eliminated from the initial sample. Since all the videos were in English, no further elimination regarding language was performed. The exclusion operation was performed by one observer, since there was little uncertainty and it was effortless during the judgment regarding to the fetched YouTube videos.

In the end, we obtained a total of 196 unique e-cigarette related YouTube videos that were coded and analyzed to achieve the goal of this study.

### Video coding

To access the overall portrayal of these e-cigarette YouTube videos, two separate reviewers rated the videos on whether they had contents that were “pro”, “anti” or “neutral” to e-cigarettes. “Pro” was defined as promoting the use of e-cigarettes, such as presenting the advantage of e-cigarettes, sharing their using experience with positive attitude toward e-cigarettes, or making them look enjoyable or socially acceptable. Videos about negative consequences of using e-cigarettes or those that contained obvious negative feedbacks or warnings were considered “anti” to e-cigarettes. Any videos that were not easily classified as either “pro” or “anti”, but included mention about e-cigarettes, were rated as “neutral” because they could make electronic cigarettes appear either positive or negative depending on the perspective of the video viewer when he/she was watching. Reviewer agreement on this rating was 95.9%.

Within each portrayal category, videos were further classified by video genres. The genres include 1) advertisement (videos created by companies to promote a specific brand or product), 2) user sharing (videos uploaded by users to share experience or tips), 3) product review (videos comparing multiple products), 4) introduction (videos introducing e-cigarettes in general), 5) celebrity use (videos showing celebrities used e-cigarettes), 6) free trial (videos featuring URL links or store address to get free products), 7) news clip (news reporting e-cigarettes) and 8) other TV program (including TV shows, interviews which focused on e-cigarettes). These genres were chosen based on recurring themes in the 196 videos.

In order to understand the detailed messages conveyed through these videos, we also examined each video to document whether there was any mention of e-cigarette promotional and warning content. Specifically, the promotional content we considered included 1) e-cigarettes are like real cigarettes, 2) using e-cigarettes are healthier than smoking, 3) e-cigarettes can help people quit smoking, 4) e-cigarettes are environmentally friendly because they will not result in second-hand smoke, 5) e-cigarettes can be used anywhere even in places where smoking real cigarettes is forbidden, 6) appearance of Web links for e-cigarettes purchase, 7) appearance of coupon code for e-cigarettes, 8) multiple flavors like strawberry, 9) using e-cigarettes will help customers save money, 10) actively demonstrating how to use e-cigarettes, 11) free trial of e-cigarettes and 12) mention of e-cigarette brands. In terms of the warning content, we considered 1) mention of health risks associated with e-cigarettes, 2) mention of US Food and Drug Administration (FDA) regulation attempts and 3) the efficacy of e-cigarettes for smoking cessation has not been proved. The above list was developed based on surveys examining motives behind e-cigarette usage and internal discussions.

Regarding the producers behind these e-cigarette videos, we classified the videos by whether they were created by a professional organization or an amateur user. If a video promoted a specific brand and had high production quality and/or if the video explicitly declared that it was produced by a professional organization, it was determined to be professional. Otherwise, the video was coded as amateur. Additionally, to gain a sense of the target audience, the demographic characteristics of the most prominent person (i.e., the messenger) in the “pro” video, such as gender and age, were estimated.

Finally, basic information was collected from each video, including the number of views, comments, favorites, likes and dislikes since YouTube users can archive videos by labeling a video as a “favorite” one and rate videos by whether they “like” or “dislike” a video. Given that YouTube provided several demographic information when the data was collected, we also documented the countries in which the video was most frequently watched, age (13–17, 18–24, 25–34, 35–44, 45–54, 55–64), and gender of those most likely to watch the video.

## Results

Among all the sample videos, 94% (n = 185) were “pro” to e-cigarettes and 4% (n = 8) were neutral, while there were only 2% (n = 3) that were “anti” to e-cigarettes. Obviously, the vast majority of the videos promoted the use of e-cigarettes. The distribution of video amount over the 8 genres is presented in Table 1. Among all the “pro” videos, 48.1% (n = 89) were advertisement. All the user sharing videos (n = 33) were “pro” ones. In the news clip genre, there were 6 videos which promoted e-cigarettes. The 3 “anti” videos were all news clips.

**Table 1:** **Genres of e-cigarette videos on YouTube**

	“pro”		“anti”		“neutral”	
	(n = 185)		(n = 3)		(n = 8)	
	#	%	#	%	#	%
Advertisement	89	48.1	0	0.0	0	0.0
User sharing	33	17.8	0	0.0	0	0.0
Product review	24	13.0	0	0.0	0	0.0
Introduction	18	9.7	0	0.0	2	25.0
News clip	6	3.2	3	100.0	6	75.0
Other TV program	7	3.8	0	0.0	0	0.0
Celebrity use	5	2.7	0	0.0	0	0.0
Free trial	3	1.6	0	0.0	0	0.0

The topics around e-cigarettes covered in the sample videos are shown in Table 2. Regarding the promotional content, in the “pro” e-cigarette videos, there were 84.3% (n = 156) videos mentioning Web links for e-cigarette purchase, which made YouTube a direct medium between e-cigarette vendors and consumers. 74.6% (n = 138) of “pro” e-cigarette videos contained scenes in which a man or woman was using e-cigarette to promote it purposefully. These scenes usually made the distinction between e-cigarette vaporing and real cigarette smoking hard to draw. Brand mentions existed in 71.9% (n = 133) of “pro” e-cigarette videos, in which “Blu”, “NJOY” and “Green Smoke” were the most frequent e-cigarette brands. As a main promotional strategy, claiming that using e-cigarettes was healthier than smoking real cigarettes existed in 71.4% (n = 132) of “pro” e-cigarette videos. Other promotion claims include using e-cigarettes can give consumers the same feeling of smoking real cigarettes (n = 80, 43.2%), e-cigarettes can be used in everywhere even in places where smoking real cigarettes was forbidden (n = 74, 40.0%) and consumers can choose from multiple flavors such as chocolate and strawberry (n = 80, 43.2%). In the “pro” e-cigarette videos, mentions of FDA regulation attempts, health risks associated with e-cigarettes and unproven efficacy for smoking cessation were only shown in 3.0% (n = 5), 1.6% (n = 3), 0.5% (n = 1) of “pro” videos, respectively.

**Table 2:** **Topics covered in e-cigarette videos on YouTube**

	“pro”		“anti”		“neutral”	
	(n = 185)		(n = 3)		(n = 8)	
	#	%	#	%	#	%
**Promotional content**						
Like real cigarettes	80	43.2	3	100.0	7	87.5
Healthier than smoking	132	71.4	0	0.0	8	100.0
Help quit smoking	52	28.1	0	0.0	4	50.0
Environmentally friendly	62	33.5	0	0.0	2	25.0
Use it anywhere	74	40.0	0	0.0	4	50.0
Web links for purchase	156	84.3	0	0.0	2	25.0
Coupon code	9	4.9	0	0.0	0	0.0
Multiple flavors	80	43.2	0	0.0	0	0.0
Save money	16	8.6	0	0.0	1	12.5
Use demonstration	138	74.6	0	0.0	3	37.5
Free trial	6	3.2	0	0.0	0	0.0
Brand mention	133	71.9	0	0.0	3	37.5
**Warning content**						
Health risks	3	1.6	3	100.0	2	25.0
FDA regulation	5	3.0	1	33.3	6	75.0
Unproven efficacy for quitting	1	0.5	0	0.0	0	0.0

Regarding the producers behind videos, 79.5% (n = 147) of the “pro” e-cigarette videos were professionally made and 20.5% (n = 38) were made by amateurs. In terms of the demographic characteristics of the most prominent person in the 172 “pro” videos in which there were specific messengers, the gender of 63.4% (n = 109) of videos’ messenger was male, while the gender of 12.2% (n = 21) of videos’ messenger was female. Both male and female messengers existed in 23.3% (n = 40) of videos. Besides, 1.2% (n = 2) of videos’ messenger was a cartoon character, which may appealed to teenagers. In terms of the estimated age of the messengers, most of them were between 25 and 35 years old.

Table 3 shows the video statistics associated with the “pro”, “anti” and “neutral” videos, which reflect the degree of viewer engagement. The 185 “pro” videos had been watched by 14,335,197 times in total and it had 56 “favorites” and 135 “likes” averagely, which were higher than “anti” and “neutral” videos. On the other hand, it had 295 “dislikes” and 360 comments for “anti” videos averagely that were much higher than “pro” videos. Preliminary examination showed that most of the comments were explicitly against the opinion that e-cigarettes were harmful to health.

**Table 3:** **Video statistics associated with e-cigarette YouTube videos**

		“pro” (n = 185)	“anti” (n = 3)	“neutral” (n = 8)
# of view	Total	14,335,197	174,638	324,486
	Average	77,488	58,213	40,561
	Range	2-2,362,588	284-90,060	8-122,256
# of comment	Total	14,746	1,080	257
	Average	81	360	37
	Range	0-2,148	0-841	0-206
# of favorite	Total	8,540	24	122
	Average	56	12	20
	Range	0-682	0-24	0-80
# of like	Total	24,092	102	234
	Average	135	34	33
	Range	0-4,430	0-58	0-133
# of dislike	Total	3,158	886	58
	Average	18	295	8
	Range	0-582	0-871	0-48

According to the demographic information available on YouTube containing information on three top gender and age groups for each video, the majority of the audience was from the “Male, 45–54 years” group. Specifically, in the 143 “pro” e-cigarette videos in which demographic information of viewers was available, “Male, 45–54 years” and “Male, 35–44 years” were the primary audience group of 92.3% (n = 132) and 90.2% (n = 129) videos, respectively. In terms of the female audience, “Female, 45–54 years” was the primary audience group of 23.1% (n = 33) of “pro” videos. Regarding the “neutral” e-cigarette videos, “Male, 35–44 years”, “Male, 45–54 years” and “Female, 45–54 years” were also the primary audience group. As a result, males and females of 45–54 years were the main audience of the sampled videos.

In terms of the geographic origins of the audience, Table 4 has shown the number of videos for each country, in which the video was popular. It is clear the viewership of these e-cigarette videos is global. However, most videos were popular in United States, United Kingdom and Canada. Specifically, in the 138 videos in which the geographic origin information for video audience was available, United States was the primary audience for 101 videos, the secondary audience for 19 videos, the tertiary audience for 6 videos.

**Table 4:** **Geographic origins of e-cigarette YouTube video audience**

	Primary		Secondary		Tertiary	
	(n = 127)		(n = 120)		(n = 110)	
	#	%	#	%	#	%
United States	101	79.5	19	15.8	6	5.5
United Kingdom	12	9.4	38	31.7	39	35.5
Canada	1	0.8	38	31.7	42	38.2
Australia	1	0.8	5	4.2	3	2.7
Hungary	1	0.8	2	1.7	3	2.7
Germany	1	0.8	1	0.8	3	2.7
Israel	0	0.0	1	0.8	3	2.7
Czech Republic	1	0.8	2	1.7	1	0.9
Slovenia	1	0.8	1	0.8	2	1.8
Finland	0	0.0	3	2.5	1	0.9
South Africa	0	0.0	2	1.7	2	1.8
Russia	1	0.8	2	1.7	0	0.0
Brazil	1	0.8	2	1.7	0	0.0
Poland	2	1.6	1	0.8	0	0.0
Philippines	1	0.8	1	0.8	0	0.0
Greece	0	0.0	2	1.7	0	0.0
Georgia	1	0.8	0	0.0	1	0.9
China	2	1.6	0	0.0	0	0.0
Thailand	0	0.0	0	0.0	2	1.8
Sweden	0	0.0	0	0.0	2	1.8

## Discussion

To the best of our knowledge, this is the first study to systematically document the quantity, portrayal and reach of e-cigarette videos on YouTube. First, we assessed the quantity of e-cigarette videos by investigating the availability of e-cigarette related messages. Through this study, we find that it is quite easy for people to get access to a larger number of e-cigarette videos. None of the sampled e-cigarette videos blocks youth viewing. Notably, males and females of 13–17 years were found among the main audience of two promotional videos on YouTube. Second, we assessed the portrayal of e-cigarette videos by investigating the overall attitude to e-cigarettes and documenting promotional and warning content for each video. This study found the vast majority of information on YouTube about e-cigarettes promoted their use and depicted the use of e-cigarettes as socially acceptable, which is agreement with previous studies of tobacco related videos on YouTube [9, 12–15, 17, 19–21]. The top 3 most prevalent genres of videos were advertisement videos produced by e-cigarette companies, user sharing videos produced by consumers and product review videos produced by vendors. Regarding the topics covered in these “pro” e-cigarette videos, most of them claimed that e-cigarette was healthier than real cigarettes and contained scenes showing that e-cigarette use was enjoyable or socially acceptable. In addition, direct Web links for e-cigarette purchase and brand mentions were very popular in these videos. On the contrary, only a tiny percent of videos mentioned facts that the efficacy of e-cigarettes for smoking cessation is not scientifically proven, some health experts are concerned about health risks associated with e-cigarette [22] and FDA is trying to regulate the production and marketing of e-cigarettes [23]. This study also found that “pro” e-cigarette videos were watched more frequently and rated much more favorably than “anti” ones. Obviously “pro” e-cigarette views were dominated in the discussion on YouTube, while “anti” e-cigarette voice was weak in the competition. Third, we assessed the reach of e-cigarette videos by investigating the number of views, comments, favorites, likes, dislikes and demographic information along with each video, which documented the countries in which the video was most frequently watched, age and gender of those most likely to watch the video. Through this study, we find males and females of 45–54 years were the main audience of the sampled videos and the viewership of these e-cigarette videos is global.

One of the most prevalent topics in the “pro” e-cigarette videos is a claim that e-cigarettes are safer and healthier alternatives to conventional cigarettes through delivering the experience of smoking while eliminating health risks associated with tobacco smoke. However, the health effects of inhaling nicotine vapor into lungs are still a subject of uncertainty and users are concerned about product safety and toxicity [24]. Currently, there is only very limited and conflicting data on toxicity, carcinogenicity and infectivity of the e-cigarette cartridges or vapors [22]. The regulatory agencies are also concerned about the quality control, since the performance of e-cigarettes is highly variable both between and within brands [25]. There is an urgent need for a better understanding of the short and long-term health effects associated with e-cigarette use.

Another highlighted topic in the “pro” e-cigarette videos, which is attractive to smokers wanting to quit smoking, is a claim that e-cigarettes can help them quit smoking. However, the WHO and FDA do not consider it to be a legitimate therapy for smokers trying to quit. Currently, their efficacy as a smoking cessation aid has yet to be adequately tested in appropriately designed trials. We do not know how effective e-cigarettes are as a smoking cessation aid and what impact does this product have on quitting. More broadly, we also need to investigate how e-cigarettes are being used (i.e., for dual use, temporary abstinence, long term as a tobacco substitute or part of a quit attempt, etc.) and by whom (i.e., covering age, socio-economic status, gender and ethnicity) [26]. Since what some e-cigarette marketers imply in the YouTube videos may mislead video viewers, further studies on the efficacy of e-cigarettes is in critical demand.

In addition to the safety and efficacy issues, public health advocates and health experts are also concerned that e-cigarettes have negative impacts on current efforts of tobacco control. First, like most of “pro” videos, e-cigarettes are presented as products like real cigarettes and their use was marketed as enjoyable or socially acceptable. To what extent, do these messages reinforce the idea of smoking? Specifically, we want to know what the impact is, if any, of e-cigarette use on the denormalisation of smoking [27]. Second, several videos say that consumers can use e-cigarettes everywhere, even in places where smoking real cigarettes is not allowed. This also raises concerns that it may undermine smoking-free legislation. Third, this study found that e-cigarette products usually come in multiple flavors (i.e., chocolate and strawberry), colors and fancy packaging and they have been endorsed by famous actors as well as fictional cartoon characters. These characteristics will appeal to adolescents and young adults. The above strategic questions are raised as e-cigarettes are becoming more and more popular. Researchers need to conduct more studies to help decision making in tobacco control.

This study found that “pro” e-cigarette information is dominated on YouTube and warning voice about the health risks associated with e-cigarette is weak. Notably, there were many disagreements along with the three “anti” e-cigarette videos. As the third most popular websites in the world, YouTube has the potential to reach and influence a huge audience. Our results suggest that it’s urgent to monitor e-cigarette videos posted on YouTube and information on other social media websites. Public health organizations should appropriately inform consumers of potential harms associated with e-cigarette use. At the same time, tobacco control advocates may also consider developing health messages to counter “pro” e-cigarette content on YouTube. In order to maximize the influence of health messages in public, in the future, it is worth studying which kind of message is the most attractive to people and whether there is any opinion leader among people.

Though currently there are no studies that examine how e-cigarette messages on YouTube may affect people’s perception, belief and behavior toward e-cigarettes, previous studies have showed that positive smoking information in movies and television can stimulate positive attitudes and beliefs as well as smoking behavior among adolescents and young adults [28]. In spite of the lack of published researches addressing whether if this association is any different, it is reasonable to anticipate a similar relationship between e-cigarette messages on YouTube and people’s perception, belief and behavior toward e-cigarettes. The presented study found that vast majority of information on YouTube about e-cigarettes promotes their use and depicts the use of e-cigarettes as socially acceptable, which can be treated as the first step toward understanding the impacts of e-cigarette YouTube videos on people.

Overall, our results demonstrate urgent need for further study of safety and efficiency of e-cigarette as tobacco cigarettes substitute. Since YouTube and other social media platforms have the ability to reach and influence a huge audience, public health community should also pay more attention on the potential impact of e-cigarette marketing on current efforts in tobacco control. Policy makers within the WHO MPOWER [29] framework also can be informed by the implications of our study. Not only careful monitoring and appropriate regulation is required, but also developing health campaigns on social media is critical for public health.

This study has several limitations. First, videos sampled in this study may differ from those sampled at a subsequent time, since YouTube videos have a rapidly changing nature. Second, we only use simple video statistics to describe the viewer engagement, instead of analyzing viewer comments along with each video in depth. Third, all the videos in the data sample were coded by only two trained reviewers and the reliability of the coding result may be affected. Last, the collected demographic data that YouTube provides may not be entirely accurate.

## Conclusions

This study presents the first surveillance studies of the portrayal of e-cigarettes on YouTube. The results presented in our study highlight the extent of “pro” e-cigarette content on YouTube. It is critical to develop appropriate health campaigns to inform e-cigarette consumers of potential harms associated with e-cigarette use. Further research is needed to evaluate the influence of e-cigarette messages in YouTube videos on people.
